# Sclerostin as a biomarker of cardiovascular risk in women with systemic lupus erythematosus

**DOI:** 10.1038/s41598-022-25651-y

**Published:** 2022-12-14

**Authors:** Carlos Garcia-de los Ríos, Marta Medina-Casado, Antonio Díaz-Chamorro, María Sierras-Jiménez, Pablo Lardelli-Claret, Rafael Cáliz-Cáliz, José Mario Sabio

**Affiliations:** 1 Department of Internal Medicine, Hospital Universitario Virgen de las Nieves, Granada, Spain; 2 Regional Blood Transfusional Center, Granada, Spain; 3 Emergency Department, Hospital Universitario Virgen de las Nieves, Granada, Spain; 4 Department of Preventive Medicine and Public Health, University of Granada, Granada, Spain; 5 Centros de Investigación Biomédica en Red de Epidemiología y Salud Pública (CIBERESP), Barcelona, Spain; 6 Instituto de Investigación Biosanitaria Ibs.GRANADA, Granada, Spain; 7 Department of Rheumatology, Hospital Universitario Virgen de las Nieves, Granada, Spain; 8 Systemic Autoimmune Diseases Unit, Department of Internal Medicine, 9th Floor, Hospital Universitario Virgen de las Nieves, Avda. Fuerzas Armadas, Nº 2, 18014 Granada, Spain

**Keywords:** Predictive markers, Risk factors, Systemic lupus erythematosus, Preclinical research, Preventive medicine, Atherosclerosis, Arterial stiffening, Carotid artery disease

## Abstract

Cardiovascular disease is one of the main causes of death in patients with systemic lupus erythematosus (SLE). On the other hand, sclerostin is a reliable and early biomarker of vascular calcification. This study aimed to estimate the association between sclerostin and two markers of cardiovascular risk, carotid atherosclerotic plaque (CP) and carotid-femoral pulse wave velocity (PWV), in women with SLE. The presence of CP (determined by carotid artery ultrasound) and PWV were measured in 68 women with SLE and preserved renal function. None of the participants had a history of cardiovascular disease. Serum levels of sclerostin were determined using the ELISA method. Other factors associated with increased cardiovascular risk were also measured. The association between sclerostin, CP and PWV was assessed using Receiver Operating Characteristic (ROC) curves and multivariate regression models. The area under the ROC curve was 0.785 (95% confidence interval [CI] 0.662–0.871) for CP and 0.834 (95% CI 0.729–0.916) for dichotomized PWV. After adjusting for other cardiovascular risk factors, it was found that a 10-units increase in sclerostin values was associated with a 44% increase in the odds of CP (95% CI 1–105), but no adjusted association was observed between sclerostin and PWV. Predictive models included age (for both outcomes), hypertension, Framingham risk score and C-reactive protein (for PWV), but not sclerostin. Sclerostin is associated with the presence of CP in women with SLE. Further research should confirm its possible role as a biomarker of cardiovascular risk in these patients.

## Introduction

Systemic lupus erythematosus (SLE) is an autoimmune disease that can damage any organ or system and usually affects women of childbearing age with a 9:1 female to male ratio^1^. There is evidence that cardiovascular disease (CVD), the leading cause of deaths in these patients^2,3^, is significantly increased in patients with SLE compared to the general population^4^. Despite the association between SLE and classical cardiovascular risk factors is well established^5–7^, the predictive value of the Framingham criteria and other conventional cardiovascular risk scores is low in these patients^8^. This can be explained by emerging cardiovascular risk factors specifically associated with SLE that seem to be crucial in the pathogenesis of atherosclerosis in autoimmune patients, and which are usually less frequent than classical cardiovascular risk factors (e.g., higher incidence in women, earlier age of presentation, or chronic inflammation)^9^. The growing knowledge on this topic has led to the proposal of mixed algorithms that combine traditional predictors with new serum biomarkers to predict CVD and identify subclinical atherosclerosis in selected cases^10^.

The presence of carotid atherosclerotic plaque (CP) determined by carotid artery ultrasound (CAU) has been extensively validated as a surrogate marker of subclinical CVD in the general population^11,12^. In addition, multiple studies have reported a higher prevalence of CP in SLE patients, as well as a more rapid progression compared to healthy controls^13–15^. On the other hand, the carotid-femoral pulse wave velocity (PWV) is increased from the onset of the atherosclerosis process^16^. Such increase, which is directly related to subclinical CVD^14^, can be non-invasively quantified using automated devices.

Sclerostin is a glycoprotein that inhibits the Wnt/β-catenin pathway, preventing osteoblast differentiation, and represents the main antagonist of bone calcification^17^. It is secreted almost exclusively by osteocytes, although its production has also been observed in other cells such as osteoclast precursors, renal and vascular cells^18^. After discovering its important anti-calcifying role, as well as its existence and production in endothelial cells, the role of sclerostin in the process of vascular calcification has been extensively studied in recent years. It is currently considered as one of the possible factors that may be included in the paradoxical regulation of the bone-vascular axis, presumably acting as an antagonist of this process^19^, and having been correlated with both clinical and subclinical CVD^20^, although with some studies with contradictory results, probably because the high blood sclerostin concentrations in participants with CVD may be a response to the disease rather than a cause^21^. In certain autoimmune diseases, such as rheumatoid arthritis, a correlation between sclerostin and arterial calcification has been observed^22^. However, the possible role of sclerostin as a marker of cardiovascular risk in women with SLE has not been studied up to date. To address this hypothesis, we designed the present study with the aim of quantifying the association between serum levels of sclerostin and two of the main cardiovascular risk markers (CP and PWV) in women with SLE.

## Methods

We analyzed a case series consisting of all women diagnosed with SLE that were consecutively attended in the outpatient clinic of the Systemic Autoimmune Diseases Unit (Department of Internal Medicine) of the Hospital Universitario Virgen de las Nieves (Granada, southern Spain) between October 2019 and March 2020. This is a tertiary-level hospital that provides specialized health care to a population of 450,000 inhabitants. The following inclusion criteria were established: meeting diagnostic criteria for SLE defined by the European Alliance of Associations for Rheumatology (EULAR) and the American College of Rheumatology (ACR)^23^, follow-up in our clinic for at least the previous year, being aged between 18 and 60 years, having an estimated glomerular filtration rate (eGFR) higher than 75 mL/min/1.73 m^2^ at the time of inclusion, a body mass index (BMI) below 40 kg/m^2^, no previously diagnosed cardiovascular events, and providing written informed consent to participate in the study. All procedures performed in the study involving human participants were in accordance with the ethical standards of the local ethics committee and with the 1964 Helsinki Declaration and its later amendments or comparable ethical standards. The study was approved by the Bioethics Committee of the Junta de Andalucia, Spain (verification code 1ec707c2e631118e6a5bc3cb1e64726137e80d0f).

In total, 68 women met these criteria and made up the final study sample. To estimate the sample size needed to achieve our primary objective, an a priori prevalence of CP among SLE around 50% was assumed based on the study by Frerix et al.^24^. To detect sensitivity and specificity values of sclerostin (as a marker of CP) equal or higher than 90% with an accuracy of 10%, 71 women with SLE would be required. Unfortunately, the outbreak of the SARS-CoV-2 pandemic at the end of our study period restricted our ability to reach this sample size.

All eligible cases who provided informed consent were scheduled for a second fasting visit in the morning (between 8 and 9 AM) in order to fulfil a questionnaire aimed to obtain clinical and demographic information. After the clinical examination, blood and urine samples were collected. Finally, the patients were scheduled for a final visit within the next 10 days to perform CAU (with the General Electric’s Logiq F6 ultrasound machine)^25^ and measurement of PWV (with the Mobil-O-Graph^®^ 24-h pulse wave analysis monitor, IEM GmbH, Stolberg, Germany)^26^. The following variables were also collected and recorded in a database: age, BMI, disease duration (in years), tobacco use, sedentary lifestyle, presence of metabolic syndrome (MetS), dyslipidemia and hypertension (HTN), eGFR, SLE Disease Activity Index (SLEDAI)^27^, and Systemic Lupus International Collaborating Clinics/American College of Rheumatology Damage Index (SDI) to measure accumulated organ damage^28^, presence of antiphospholipid antibodies (ELISA), serum levels of sclerostin (measured by the bioactive Sclerostin ELISA kit, BI-20472) and specific treatments for SLE (grams of prednisone taken in the past year and use of hydroxychloroquine). Blood pressure (BP) was measured in duplicate, 5 min apart, in the dominant arm with the patient seated and after at least 5 min of rest, using a validated automatic oscillometric device (HEM-7051T; Omron Health Care, Kyoto, Japan). The lowest value of the two measurements taken was considered for the study. BMI was calculated as the weight in kilograms divided by height in meters squared. Dyslipidemia was defined as a low-density lipoprotein cholesterol (LDLc) level above 130 mg/dL. The definitions of HTN, obesity, smoking, and sedentary lifestyle have been previously described elsewhere^29^. MetS was defined according to the criteria of the National Cholesterol Education Program Adult Treatment Panel III^30^. Finally, eGFR was calculated using the Modification of Diet in Renal Disease (MDRD)-7 equation.

Carotid plaques were defined as any focal protrusion into the arterial lumen of thickness > 0.5 mm or > 50% of the surrounding intima-media thickness or a diffuse thickness > 1.5 mm measured between the media-adventitia and intima-lumen interfaces^12^. PWV was expressed in meters per second.

### Data analysis

First, differences in the distribution of sclerostin, PWV and the remaining study variables were evaluated according to CP status. The Receiver Operating Characteristic (ROC) curve of PWV values as a marker of CP was obtained to estimate the area under the curve (AUC) and the best cut-off point of the PWV values. The same procedure was applied to assess the relationship between sclerostin levels and both CP and PWV values (using the previously defined cut-off point). Finally, two regression models were adjusted to estimate the relationship between sclerostin and the two cardiovascular outcomes: a logistic model for CP and a linear model for PWV values. The former allowed the estimation of the Odds Ratio (OR) as a measure of association; and the latter provides the corresponding regression coefficient. Both models were applied in three steps: first, univariate models were constructed for each independent variable to estimate crude measures of association between the two outcomes variables; second, multivariate explanatory models were adjusted, including all study variables as independent terms in the models; finally, predictive models were adjusted using a stepwise forward procedure, according to which only the independent terms with p values below 0.05 were included in the model, while those with p values higher than 0.1 were excluded. All analyses were performed with the Stata statistical package (version 17)^31^.

## Results

### Differences between women with SLE with and without CP

Carotid plaques were present in 24 of the 68 women (35.3%). Table 1 shows the descriptive statistics for the main study variables in the whole sample and separately in women with and without CP. The mean value of PWV was 6.4 m/s (SD: 1.2), and was significantly higher in patients with CP (7.4 vs 5.9 m/s; p < 0.001). The mean value of sclerostin in the entire sample was 69.1 U/L, with significant differences depending on the presence or absence of CP (96.6 vs 54.1 U/L, respectively, p < 0.001). Other cardiovascular risk predictors directly related to CP with significant or almost significant values were age (p < 0.001), disease duration (p = 0.003), HTN (p = 0.053), presence of MetS (p = 0.066), serum levels of C3 (p = 0.016), and the Framingham risk score (p < 0.001).

**Table 1:** Distribution of study variables in the whole sample and according to CP status.

Variable	Total sample		Presence of CP				P value^d^
			Yes (n = 24)		No (n = 44)		
	Mean/n^a^	SD/%^b^	Mean/n^a^	SE/%^c^	Mean/n^a^	SE/%^c^	
PWV^e^ (m/s)	6.4	1.2	7.4	0.21	5.9	0.15	< 0.001
Sclerostin^e^ (U/L)	69.1	10.2	96.6	9.3	54.1	5.0	< 0.001
Age^e^ (years)	43.8	11.0	52.2	1.2	39.3	1.6	< 0.001
BMI^e^ (kg/m^2^)	25.6	4.9	26.4	1.2	25.2	0.61	0.359
SLE duration (years)	15.5	8.3	19.4	1.4	13.3	1.2	0.003
Smoking (n, %)	16	23.5	6	25.0	10	22.7	0.833
Sedentary (n. %)	34	50.0	13	54.2	21	47.7	0.612
HTN (n, %)	29	42.7	14	58.3	15	34.1	0.053
LDL ≥ 130 (mg/dL) (n, %)	24	35.3	11	45.8	13	29.6	0.179
MetS (n, %)	12	17.7	7	29.2	5	11.4	0.066
SLEDAI-SELENA^f^	0	0–2	0	0–2	1	0–2	0.084^g^
SDI = 1 (n, %)	13	19.1	2	8.3	11	25.0	0.240
SDI = 2 (n, %)	3	4.4	1	4.2	2	4.6	
APS (n, %)	6	8.8	3	12.5	3	6.8	0.430
Prednisone^h^ (g)	0.86	1.3	0.68	0.25	0.96	0.20	0.400
HCQ use (n, %)	58	85.3	20	83.3	38	86.4	0.736
LN (n, %)	30	44.1	8	33.3	22	50.0	0.186
CPR (mg/dL)	3.31	6.5	5.2	1.9	2.3	0.7	0.083
C3 (mg/dL)	82.36	20.8	90.4	5.0	77.9	2.6	0.016
C4 (mg/dL)	14.6	6.0	16.0	1.5	13.9	0.7	0.176
Framingham^f^	1	1–4	4	2–6	1	0–1	< 0.001^g^

*CP* carotid plaque, *SD* standard deviation, *SE* standard error, *PWV* pulse wave velocity, *BMI* body mass index, *HTN* hypertension, *LDL* low density lipoprotein, *MetS* metabolic syndrome, *SLEDAI-SELENA* Systemic Lupus Erythematosus Disease Activity Index, *SDI* Systemic Lupus International Collaborating Clinics/American College of Rheumatology Damage Index, *APS* antiphospholipid syndrome, *HCQ* hydroxychloroquine, *LN* lupus nephritis, *CPR* C-reactive protein.

^a^Mean values (for continuous variables)/number of subjects (for categorical variables); ^b^SD (for continuous variables)/%: percent of cases in the sample (for categorical variables); ^c^SE (for continuous variables)/%: percent of cases in each category of CP (for categorical variables); ^d^p values of Student’s t test for independent samples (for continuous variables), p values of chi square test (for categorical variables); ^e^Continuous variables; ^f^Median and interquartile range is shown for these variables; ^g^p value of Mann–Whitney *U* test; ^h^Glucocorticoids accumulated during the last year.

### Relationship between PWV and sclerostin with CP status

**Figure 1:**
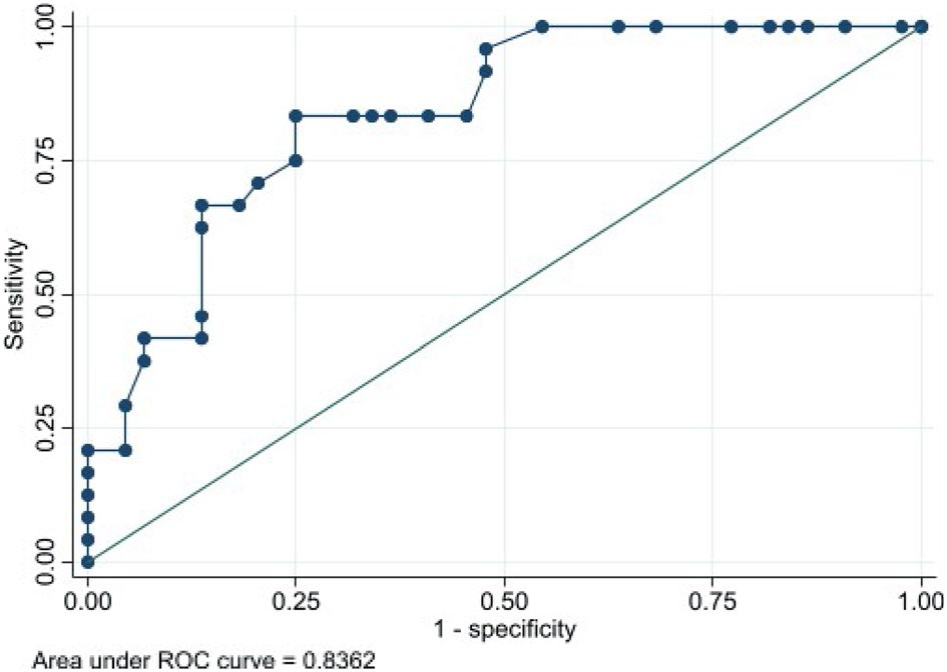
ROC curve and AUC for the values of PWV versus CP status.

**Figure 2:**
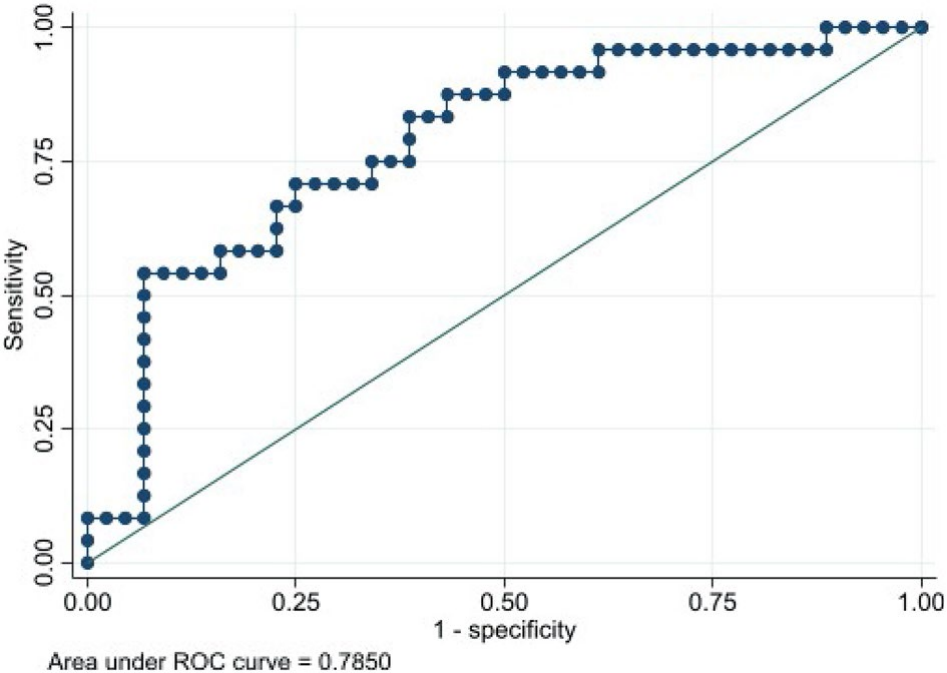
ROC curve for the values of sclerostin versus CP status.

**Figure 3:**
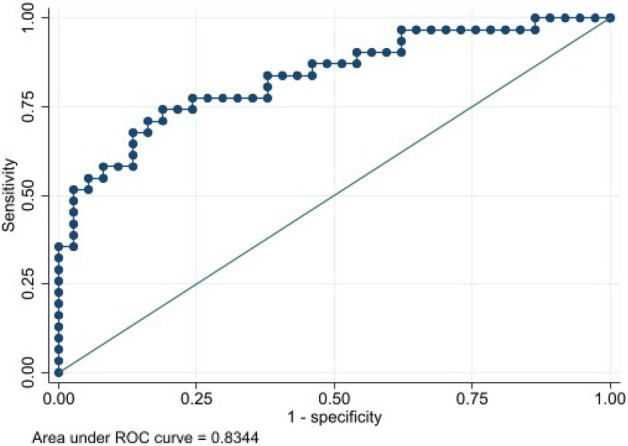
ROC curve for the values of sclerostin versus PWV dichotomized into values below or equal/above than 6.7 m/s.

Figure 1 shows the ROC curve for the values of PWV versus CP status. An AUC of 0.836 (95% CI 0.729–0.916) was obtained. The cut-off point of PWV that maximized its classification accuracy regarding CP status was 6.7 m/s, with a sensitivity value of 83.3% (95% CI 62.6–95.3) and a specificity value of 75.0% (95% CI 59.7–86.8). Figure 2 shows the ROC curve for the values of sclerostin versus CP status. An AUC of 0.785 (95% CI 0.662–0.871) was obtained. The cut-off point of sclerostin that maximized its classification accuracy regarding CP status was 99.2 U/L, with a sensitivity value of 54.2% (95% IC 32.8–74.4) and a specificity value of 93.2% (95% CI 81.3–98.6). Finally, Fig. 3 shows the ROC curve for the values of sclerostin versus PWV dichotomized into values below or equal/above 6.7 m/s. An AUC of 0.834 (95% CI 0.729–0.916) was obtained. The cut-off point of sclerostin that maximized its classification accuracy regarding dichotomized PWV was 62.9 U/L, with a sensitivity value of 75.8% (95% CI 57.7–88.9) and a specificity value of 81.1% (95% CI 64.8–90.4).

### Logistic models applied to CP as the dependent variable

Table 2 summarizes the results of the logistic models applied to CP as the dependent variable. The crude OR estimate for sclerostin (× 10 units) was 1.31 (95% CI 1.13–1.53), indicating a 31% increase in the odds of CP being present for each 10-units increase in sclerostin values. Other independent variables related to significant increases in the odds of CP were age, disease duration and the Framingham risk score. In the multivariate explanatory model (which included all independent variables), the adjusted OR estimate for sclerostin yielded a slightly higher significant value (OR 1.44; 95% CI 1.01–2.05). In the predictive model, the stepwise procedure only allowed for the inclusion of age, with a significant 19% increase in the odds of CP for each 1-year increment in age.

**Table 2:** Odds ratio estimates obtained through logistic regression models for the presence of CP.

Variables	Crude estimates		Adjusted estimates^a^		Predictive model^b^	
	OR	95% CI	OR	95% CI	OR	95% CI
Sclerostin^c^	1.31	1.13; 1.53	1.44	1.01; 2.05		
Age	1.19	1.09; 1.30	1.16	0.90; 1.50	1.19	1.09; 1.30
BMI	1.05	0.95; 1.16	0.67	0.46; 0.96		
SLE duration	1.10	1.03; 1.19	1.06	0.91; 1.25		
Smoking	1.13	0.35; 3.62	0.02	0.00; 1.92		
Sedentary	1.29	0.48; 3.51	0.44	0.05; 3.84		
HTN	2.71	0.97; 7.53	0.71	0.08; 6.68		
LDL ≥ 130 mg/dL	2.02	0.72; 5.66	2.34	0.18; 29.79		
MetS	3.21	0.89; 11.57	25.12	0.36; 1737		
SLEDAI-SELENA	0.81	0.60; 1.07	0.51	0.18; 1.45		
SDI > 0	0.34	0.09; 1.34	0.03	0.00; 0.86		
APS	1.95	0.36; 10.52	3.37	0.06; 186.88		
Prednisone	0.84	0.55; 1.27	0.87	0.31; 2.45		
HCQ use	0.79	0.20; 3.13	1.27	0.03; 54.47		
LN	0.50	0.18; 1.41	1.40	0.04; 48.04		
CPR	1.07	0.97; 1.19	1.10	0.85; 1.42		
C3	1.03	1.00; 1.06	0.93	0.85; 1.01		
C4	1.06	0.97; 1.16	1.17	0.85; 1.60		
Framingham	1.69	1.29; 2.22	2.02	0.78; 5.29		

*BMI* body mass index, *HTN* hypertension, *LDL* low density lipoprotein, *MetS* metabolic syndrome, *SLEDAI-SELENA* Systemic Lupus Erythematosus Disease Activity Index, *SDI* Systemic Lupus International Collaborating Clinics/American College of Rheumatology Damage Index, *APS* antiphospholipid syndrome, *HCQ* hydroxychloroquine, *LN* lupus nephritis.

^a^The model included all the independent variables shown in the table.

^b^The stepwise model included only variables with a p-to-enter value < 0.05 and a p-to-remove value > 0.10.

^c^Estimated for sclerostin value × 10.

### Logistic models applied to PWV as the dependent variable

Table 3 displays the regression coefficients of the linear models adjusted for PWV values as the dependent variable. The crude estimate reveals a significant positive association for sclerostin: every 10-units increase was associated with 0.14-units of increase in PWV values (95% CI 0.08–0.20). Age, BMI, disease duration, HTN, LDL values higher than 130 mg/dL, MetS, serum levels of C3 and the Framingham risk score were also positively related to PWV values. In contrast, SLEDAI and lupus nephritis exhibited an inverse relationship with PWV. The adjusted regression coefficient for sclerostin was − 0.03 (95% CI − 0.06 to 0.00). The stepwise model included age, HTN and the Framingham risk score (with positive coefficients), and C-reactive protein (with a negative coefficient), as significant independent predictors of PWV values.

**Table 3:** Regression coefficients obtained through linear regression models for PWV values.

Variable	Crude estimates		Adjusted estimates^a^		Predictive model^b^	
	Coeff	95% CI	Coeff	95% CI	Coeff	95% CI
Sclerostin^c^	0.14	0.08; 0.20	− 0.03	− 0.06; 0.00		
Age	0.10	0.09; 0.11	0.08	0.06; 0.10	0.08	0.07; 0.09
BMI	0.11	0.05; 0.16	− 0.01	− 0.04; 0.02		
SLE duration	0.06	0.03; 0.10	0.02	− 0.00; 0.03		
Smoking	0.44	− 0.27; 1.15	0.16	− 0.18; 0.50		
Sedentary	0.49	− 0.11; 1.08	− 0.04	− 0.29; 0.21		
HTN	1.05	0.49; 1.61	0.35	0.10; 0.60	0.29	0.08; 0.51
LDL ≥ 130 mg/dL	0.88	0.29; 1.48	− 0.13	− 0.41; 0.14		
MetS	1.33	0.60; 2.05	0.33	− 0.11; 0.78		
SLEDAI-SELEN	− 0.15	− 0.30; − 0.02	0.00	− 0.06; 0.06		
SDI > 0	− 0.10	− 0.81; 0.62	0.04	− 0.23; 0.30		
APS	0.29	− 0.77; 1.36	0.13	− 0.26; 0.52		
Prednisone	− 0.02	− 0.25; 0.22	0.06	− 0.04; 0.16		
HCQ use	− 0.34	− 1.19; 0.52	− 0.38	− 0.72; − 0.04		
LN	− 0.61	− 1.20; − 0.01	− 0.39	− 0.71; − 0.06		
CPR	0.03	− 0.02; 0.07	− 0.03	− 0.05; − 0.01	− 0.02	− 0.04; − 0.01
C3	0.02	0.01; 0.03	− 0.00	− 0.01; 0.01		
C4	0.04	− 0.01; 0.09	0.02	− 0.01; 0.04		
Framingham	0.39	0.33; 0.46	0.10	0.03; 0.17	0.12	0.07; 0.18

*BMI* body mass index, *HTN* hypertension, *LDL* low density lipoprotein, *MetS* metabolic syndrome, *SLEDAI-SELENA* Systemic Lupus Erythematosus Disease Activity Index, *SDI* Systemic Lupus International Collaborating Clinics/American College of Rheumatology Damage Index, *APS* antiphospholipid syndrome, *HCQ* hydroxychloroquine, *LN* lupus nephritis.

^a^The model included all the independent variables shown in the table.

^b^The stepwise model included only variables with a p-to-enter value < 0.05 and a p-to-remove value > 0.10.

^c^Estimated for sclerostin value × 10.

## Discussion

Our results suggest for the first time the existence of an unadjusted association between sclerostin and two of the main cardiovascular risk markers (CP and PWV) in patients with SLE. In addition, this association was maintained in CP in the adjusted models. These results are in line with the possible relationships between sclerostin concentrations and cardiovascular morbidity and mortality, especially in patients with chronic kidney disease, but also in diabetics and in the general population^20,32–34^. Moreover, prospective studies have demonstrated a possible relationship between serum sclerostin levels and CVD^34,35^. Regarding subclinical CVD, it has been shown that serum concentrations of sclerostin may be a good biomarker to identify patients with established subclinical vascular calcification. In fact, sclerostin has been found to be an independent marker of increased PWV^36,37^, presence of CP^38^, and to be significantly associated with aortic calcification^39^.

There is no current evidence on the possible role of sclerostin as a marker of cardiovascular risk in SLE patients. Only one previous study by Fayed et al. showed an increase in the serum concentration of sclerostin in patients with SLE compared to healthy subjects, as well as an independent relationship with proteinuria levels^40^. This latter association prompted us to exclude cases of SLE with chronic kidney disease in order to avoid its possible role as a confounding factor in the association between sclerostin and cardiovascular risk.

The reason why SLE is such an important risk factor for atherosclerosis and CVD has not yet been fully explained. The constant stimulation due to long-lasting systemic inflammation, as well as the chronic use of treatments such as corticosteroids and other factors typical of this disease, could all contribute to the formation and disruption of atherosclerotic plaques^9^. In addition, a higher incidence of classical cardiovascular risk factors like HTN or dyslipidemia is present in these patients^5,10,29^, as has been corroborated in our sample. On the other hand, there is ample evidence that the cardiovascular risk in the target population in which SLE occurs has been systematically underestimated in clinical practice. Therefore, these patients have received an under-therapeutic attitude with respect to male populations with the same characteristics^41^. For all these reasons, traditional cardiovascular risk scores have failed on their own in the early and effective identification of high-risk patients and it has been recommended in the latest cardiovascular risk guidelines to consider CVD risk assessment in patients with any chronic inflammatory condition^42^. In our sample, the Framingham score has been shown to be an independent and even predictive factor of subclinical cardiovascular risk. Thus, it should be considered in the evaluation of these patients, increasing, along with other factors, the positive predictive value of new algorithms of cardiovascular risk screening. On the other hand, markers of inflammation and scales of activity or cumulative damage have not been related to subclinical CVD.

According to the AUCs values estimated in our study, the accuracy of sclerostin as a marker of both CP and dichotomized PWV seems acceptable, especially with regard to its specificity. To our knowledge, no previous work in the literature compared the AUC of sclerostin with these outcome variables in other populations. The results of univariate models agree with the AUC values. Furthermore, despite not reaching the statistically required sample, we have observed a significant and independent relationship between sclerostin and CP after adjusting for the rest of the classical risk factors. However, although sclerostin maintained the magnitude of its association with CP after adjustment, this was not the case for PWV. This may be due to intrinsically different mechanisms of CP formation in SLE patients compared to other populations with high cardiovascular risk. For instance, a previous study found an abnormally high prevalence of CP without an associated increase in the intima-media thickness^24^. Finally, predictive models did not include sclerostin as an independent predictive marker for either CP or PWV. Age is by far the strongest predictor for both outcomes in our sample.

Some limitations should be taken into account for a correct interpretation of our results. First, our sample does not cover the true spectrum of SLE patients. As previously noted, women with chronic kidney disease were not included in the study, which implies a lower prevalence of CVD in the sample that, in turn, could have led to an underestimation of the association between sclerostin and cardiovascular risk. Likewise, men were excluded from the study due to the low prevalence of this disease in the male population. In fact, the final prevalence of CP found in our case series (35.3%) was lower than that used to estimate our sample size (i.e., 50%). This fact, together with not having been able to reach the prespecified sample size, reduced our capacity to include sclerostin in the predictive models of cardiovascular risk. On the other hand, our study only included an isolated measurement of serum sclerostin levels, without taking into account other factors that can modify its concentration, such as vitamin D or parathormone. Serial measurements of both PWV and sclerostin levels are necessary to establish true clinical utility.

In conclusion, our results reveal a significant association between sclerostin levels and CP and, to a lesser extent, PWV. This finding suggests that sclerostin could be tested as a serum marker in routine medical practice to screen and facilitate the detection of potential candidates for CAU. Further studies of SLE patients with larger sample sizes and prospectively followed-up should be carried out to confirm the clinical utility of sclerostin in routine practice.

## Data Availability

The datasets that support the findings of this study are available from the corresponding author on reasonable request.
